# Supplementary figures and images for: Viral protein instability enhances host-range evolvability
Source: PLoS Genet. 2022 Feb 17;18(2):e1010030. doi: 10.1371/journal.pgen.1010030 (PMC8890733; doi:10.1371/journal.pgen.1010030)

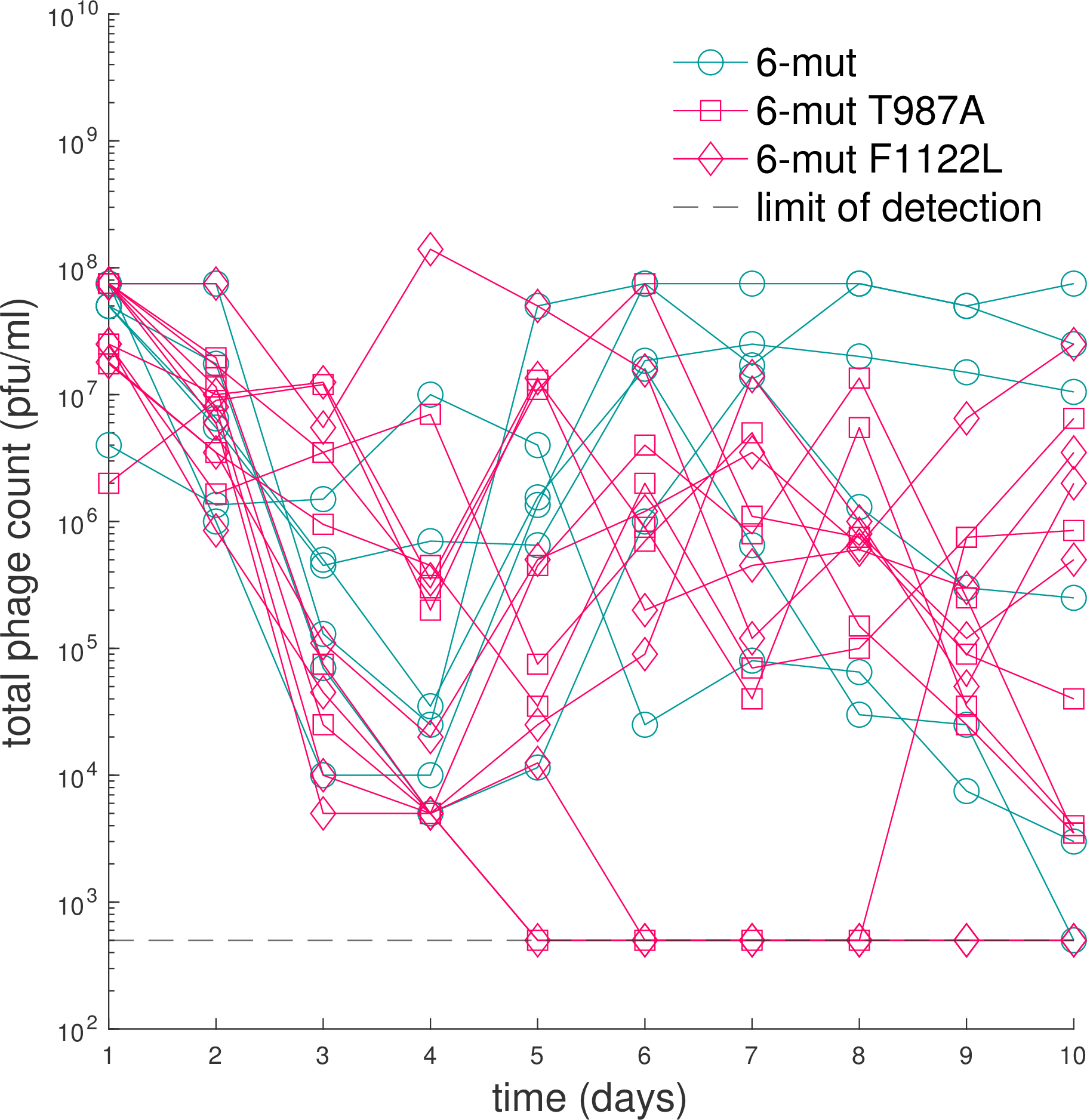

Supplement: S1 Fig — Phage titer measured on WT cells on each day of the evolution experiment on the naturally evolved thermostable variants (Fig 2). (TIF) [file pgen.1010030.s001.tif]

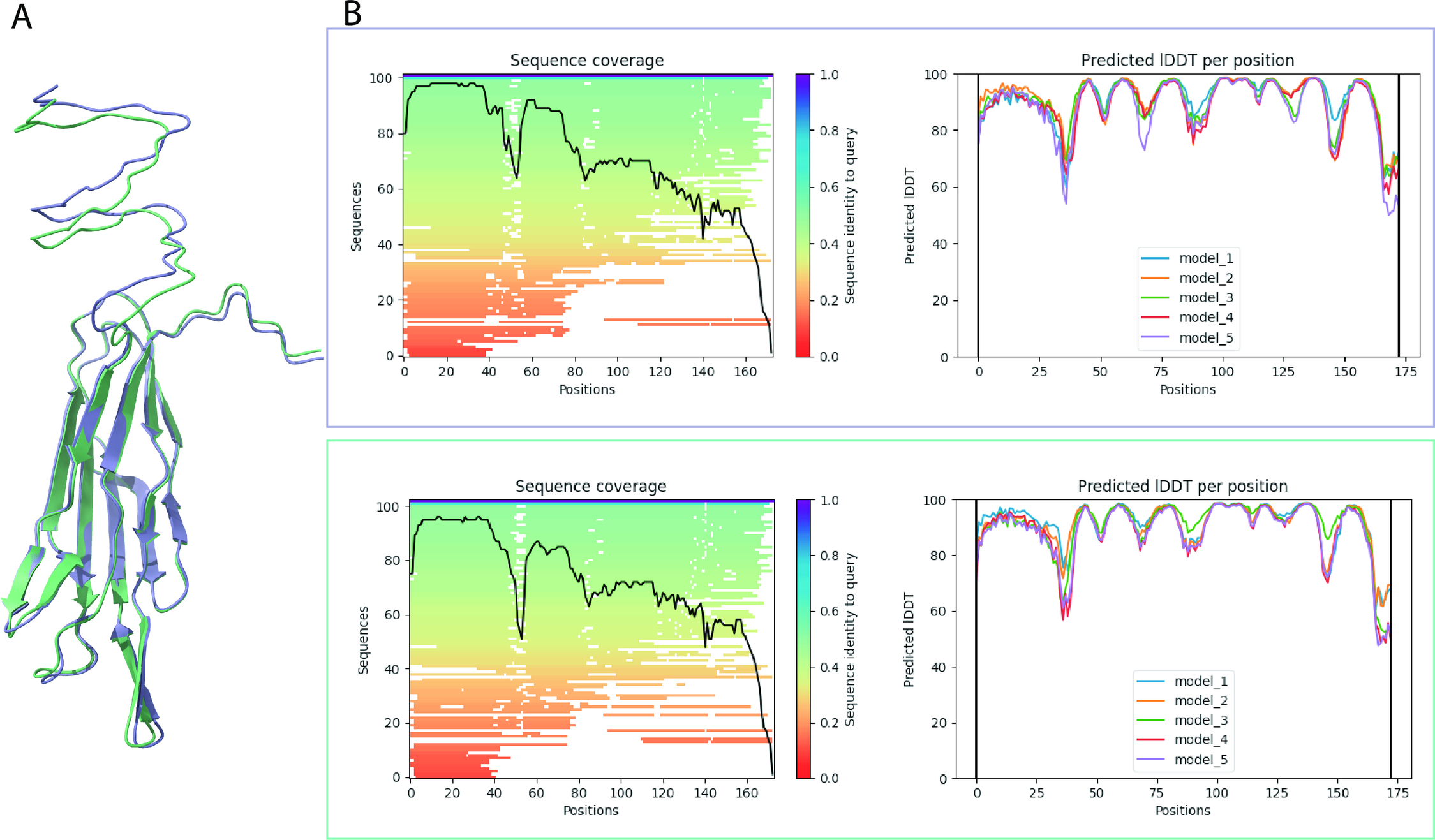

Supplement: S2 Fig — A: aligned Alphafold predictions for ancestor (purple) and 6-mut (teal) J protein reactive region (amino acids 960–1132). B: Coverage of multiple sequence alignment (MSA) used to make structural prediction and predicted IDDT (model confidence out of 100) at each position. (TIF) [file pgen.1010030.s002.tif]

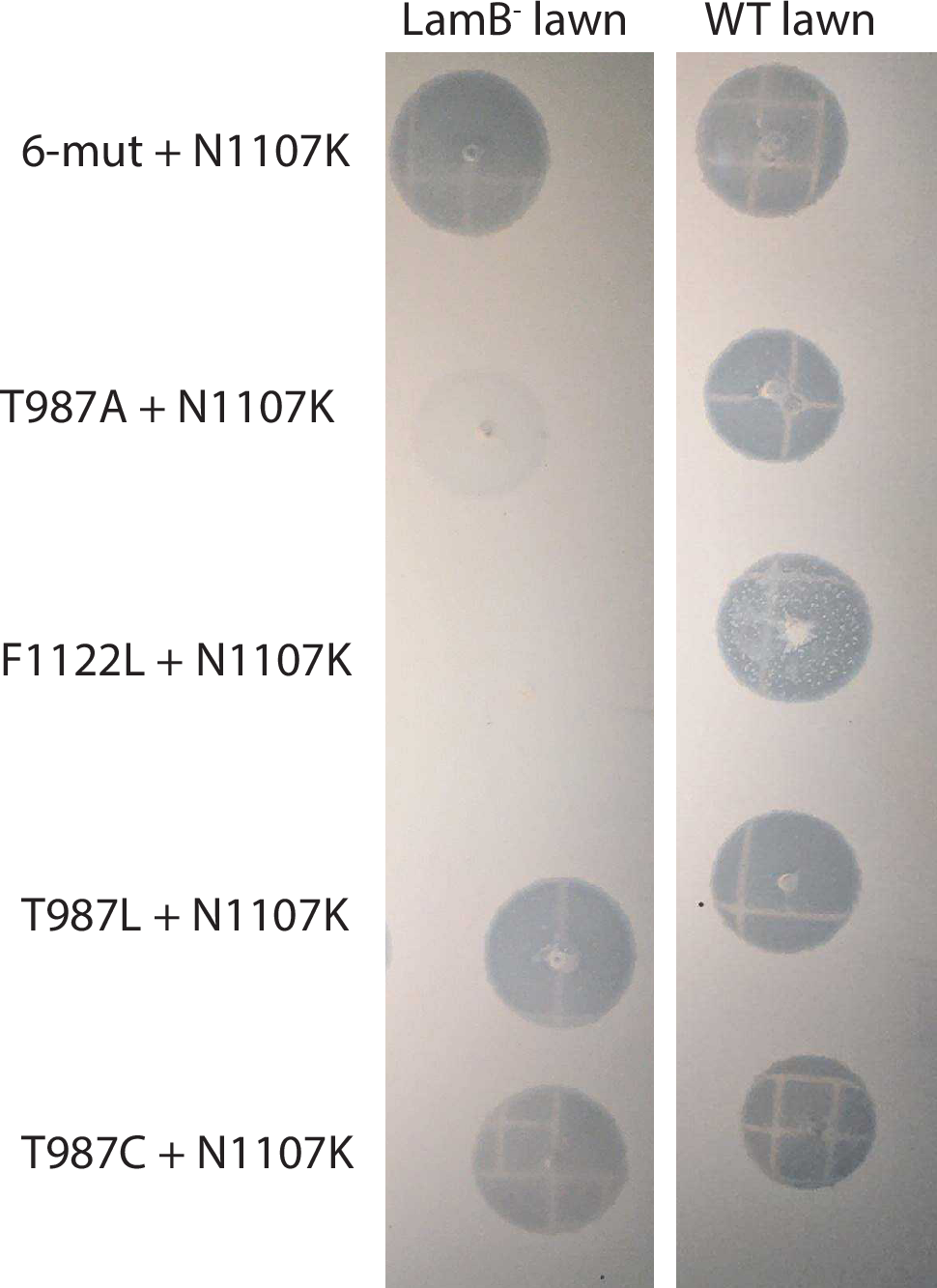

Supplement: S3 Fig — To verify that most of the stable genotypes required an additional mutation along with N1107K for OmpF+, we edited N1107K back into all genotypes in the library (S1 Text) and tested their ability to plaque on LamB—. N1107K conferred full OmpF+ on three genotypes (6-mut, T987L, and T987C), and these were all detected during the evolution experiment. Two variants, T987A and T987Y, were able to form very turbid clearings when spotted on LamB—, but because these genotypes were not able to form individual plaques we did not detect them during the evolution experiment. Consistent with their poor plaquing, genotypes with partial OmpF-use grew at a dramatically lower rate compared to genotypes with full OmpF-use (S4 Fig). All genotypes were spotted on WT lawns as a positive control to indicate that plaquing effect was specific to OmpF-use and not indicative of generic viability. (TIF) [file pgen.1010030.s003.tif]

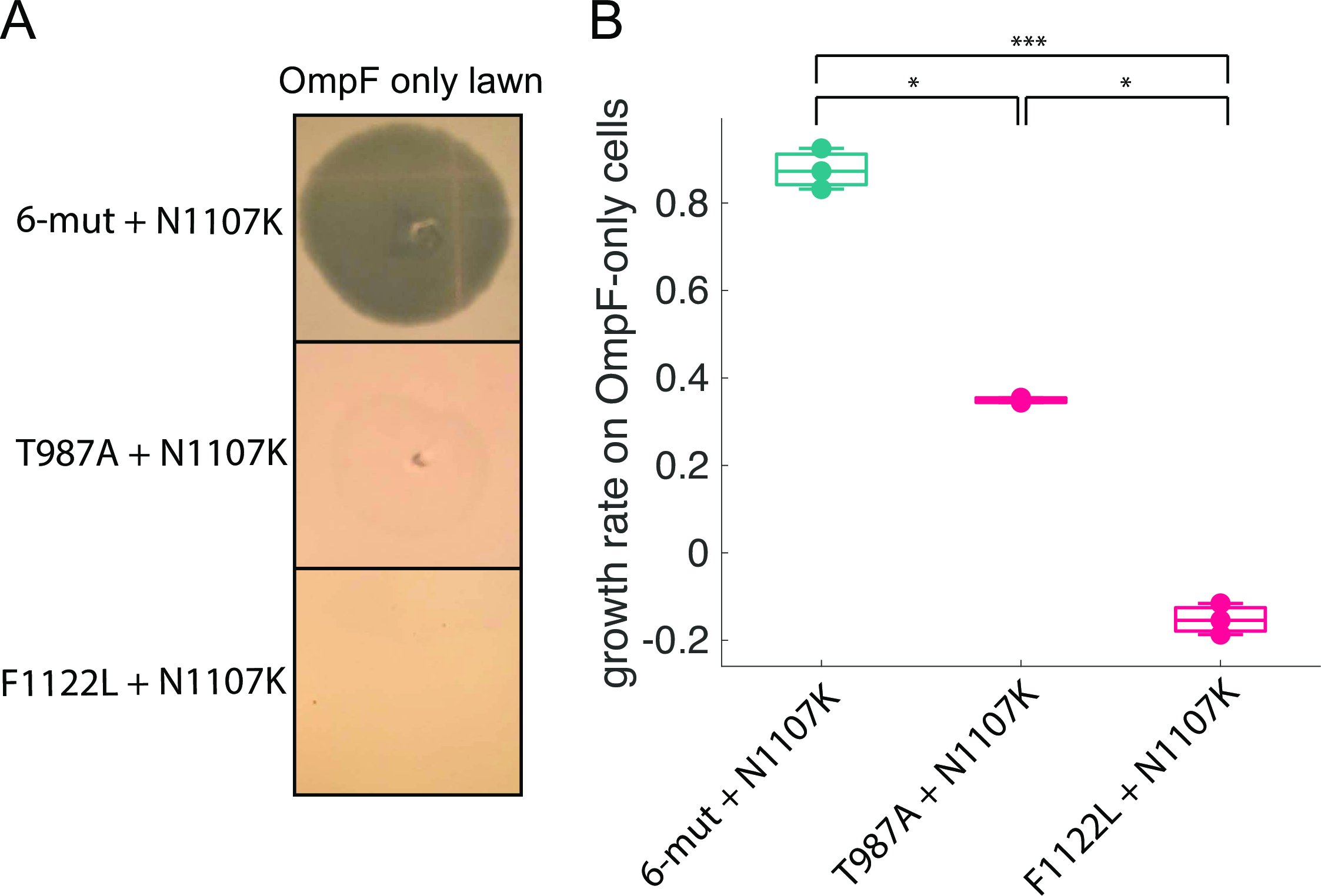

Supplement: S4 Fig — A: Plate photo of each genotype spotted on a lawn of cells that express OmpF only.B: Growth rate of three replicates of each genotype on cells that express OmpF but not LamB (n = 3 for 6-mut N1107K and F1122L N1107K, n = 2 for T987A N1107K; paired t-tests corrected for multiple comparisons using Bonferroni method; 6-mut + N1107K vs. 6-mut T987A + N1107K: p = 6.38x10^-4^; 6-mut + N1107K vs. 6-mut F1122L + N1107K: p = 3.38x10^-4^; 6-mut + N1107K vs. 6-mut F1122L + N1107K: p = 1.06x10^-6^. Bonferroni adjusted significance: ns: > 0.0167, *: p < 0.00167, **: p < 1.67e-4, *** p < 1.67e-5, ****: p < 1.67e-6.) (TIF) [file pgen.1010030.s004.tif]

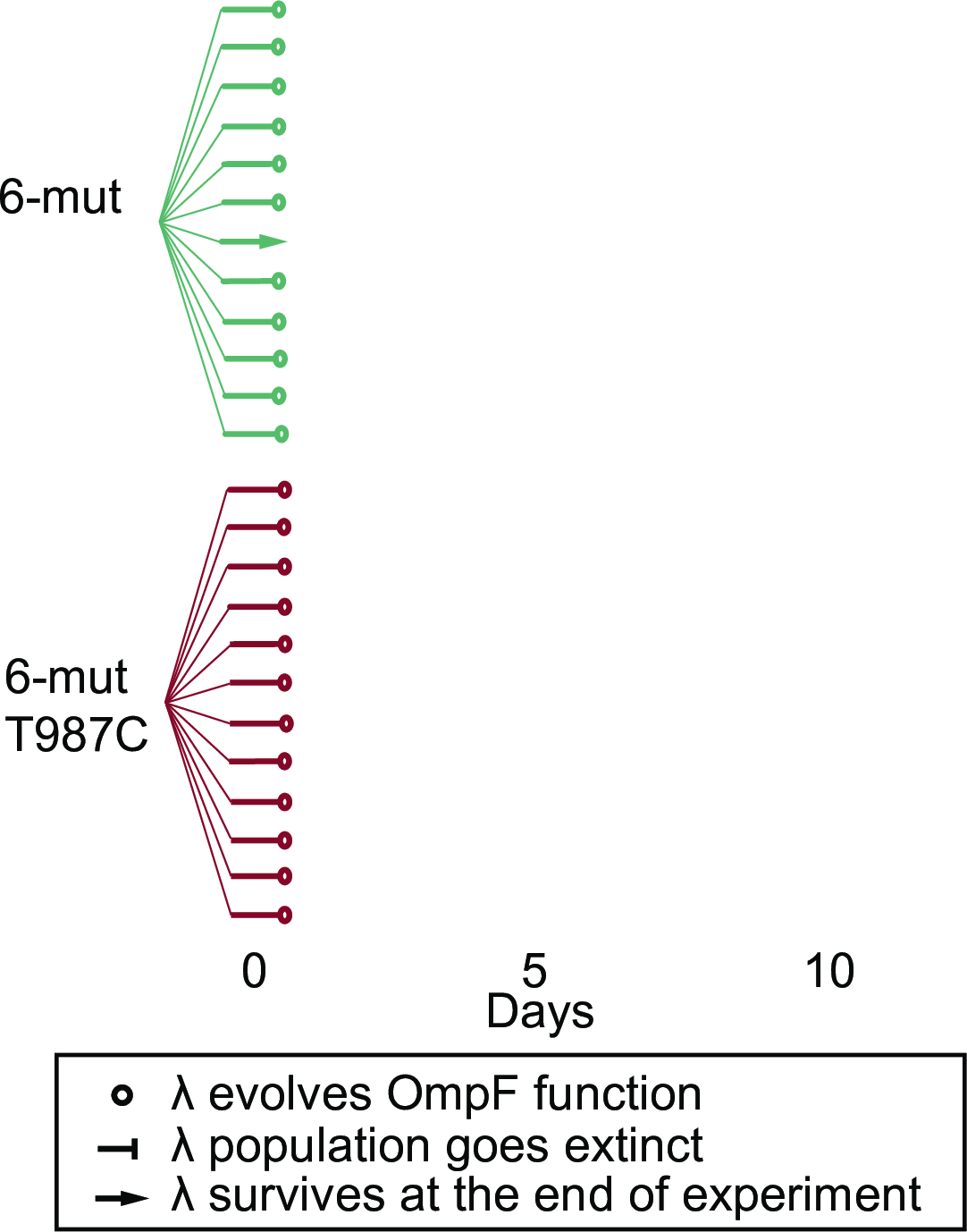

Supplement: S5 Fig — To rule out the possibility that T987C had acquired mutations outside of J that helped it evolve OmpF+ faster, we re-engineered a new lysogen with an oligo specifically designed to produce T987C. We then measured its evolvability in 12 replicate populations, as well as 12 replicate populations of the 6-mut as a control. All 12 T987C replicate populations evolved OmpF+ in one day, compared to eleven of twelve 6-mut replicate populations, confirming that these genotypes have nearly identical evolvabilities under the conditions used in our evolution experiments. (TIF) [file pgen.1010030.s005.tif]

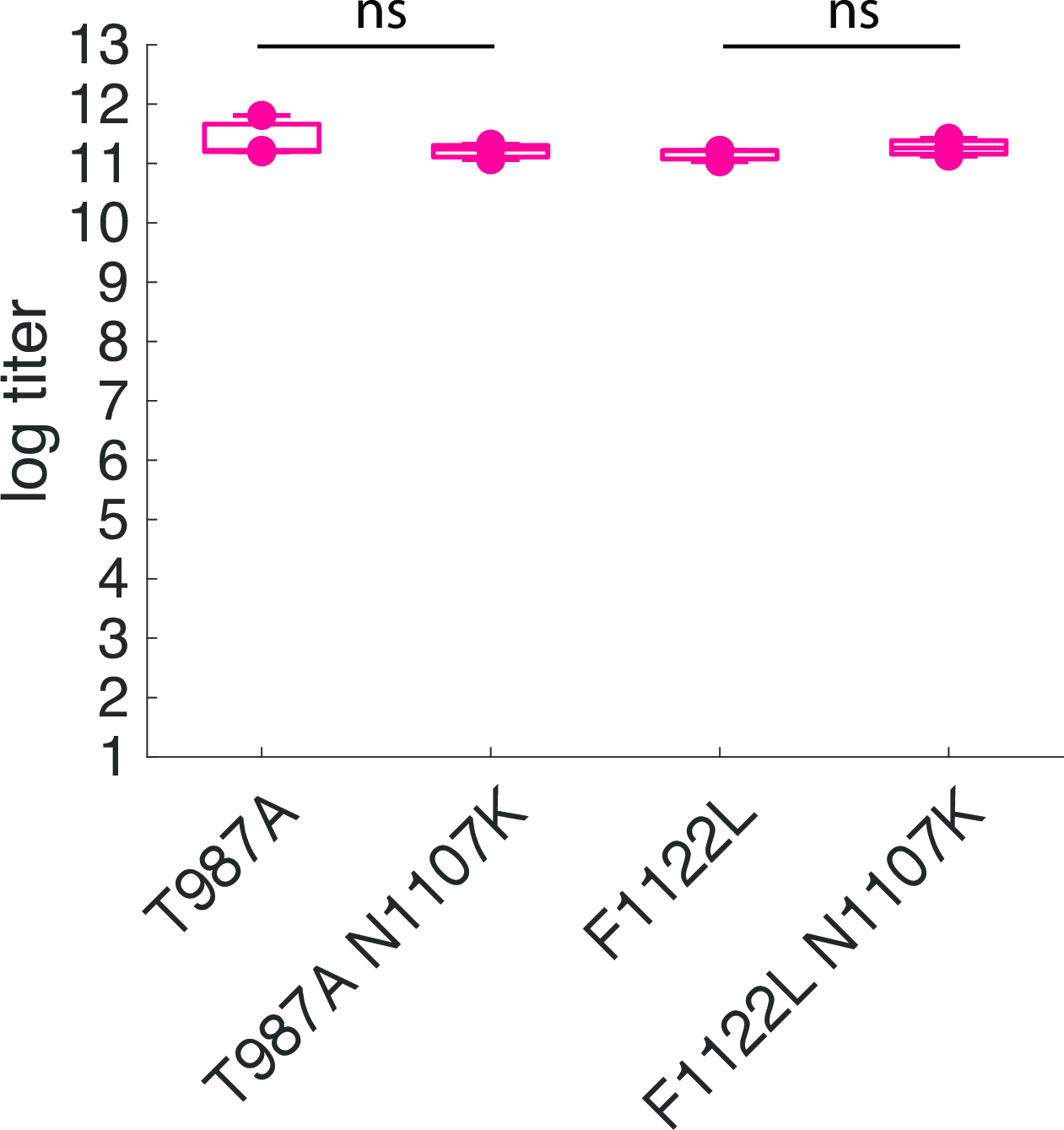

Supplement: S6 Fig — Productivity (log viable titer) of each genotype immediately after lysogen induction, with and without N1107K mutation. Statistical comparisons were made using two t-tests and corrected for multiple comparisons using the Bonferroni method, N = 3 per genotype. 6-mut T987A vs. 6-mut T987A N1107K: p = 0.4376, 6-mut F1122L vs. 6-mut F1122L N1107K: p = 0.3508. Bonferroni adjusted significance: ns: p > 0.025. (TIF) [file pgen.1010030.s006.tif]
