# Supplementary material for: Viral protein instability enhances host-range evolvability
Source: PLoS Genet. 2022 Feb 17;18(2):e1010030. doi: 10.1371/journal.pgen.1010030 (PMC8890733; doi:10.1371/journal.pgen.1010030)
Supplement: S1 Table — 6-mut was included as a positive control because it is expected to convert to OmpF+ upon receiving the N1107K mutation. As an additional positive control to ensure that the MAGE process was working for each variant, we used an oligo mix containing N1107K as well as the appropriate galK conversion oligo and computed the fraction of cells that were successfully converted from galK+ to galK—or vice versa. We computed the conversion rate to OmpF+ after MAGE by inducing the lysogens and plating dilutions of each lysate on two lawns: one with cells expressing only OmpF, and another with WT cells. We then divided the number of plaques on the OmpF only lawn by the total number of plaques on the WT lawn to get the conversion rate. In T987S, T987K, T987R, and T987Y we did not observe any OmpF+ plaques, so we computed an upper bound as the conversion rate if a single OmpF+ plaque had been observed. For T987A, F1122L, T987L and T987C, we separately engineered in N1107K and isolated and sequenced individual clones rather than using the high throughput method described here. (DOCX) [file pgen.1010030.s007.docx]

| genotype | positive control (*galK*) conversion rate | conversion rate to OmpF+ by N1107K oligo |
| --- | --- | --- |
| 6-mut | 6.20E-02 | 2.00E-01 |
| 6-mut | 9.04E-02 | 1.85E-01 |
| 6-mut | 6.54E-02 | 9.38E-02 |
| T987S | 9.49E-02 | <3.20E-07 |
| T987S | 1.07E-01 | <3.60E-07 |
| T987S | 1.20E-01 | <6.40E-07 |
| T987G | 4.96E-02 | <8.00E-04 |
| T987G | 2.86E-02 | <1.30E-03 |
| T987G | 5.36E-02 | <2.70E-03 |
| T987K | 5.01E-02 | <1.10E-06 |
| T987K | 5.31E-02 | <1.10E-06 |
| T987K | 5.04E-02 | <4.00E-05 |
| T987R | 4.93E-02 | <1.60E-06 |
| T987R | 6.24E-02 | <3.20E-06 |
| T987R | 6.90E-02 | <3.20E-06 |
| T987Y | 3.48E-02 | <1.10E-06 |
| T987Y | 3.10E-02 | <4.00E-07 |
| T987Y | 3.47E-02 | <6.40E-07 |
