# Supplementary material for: Viral protein instability enhances host-range evolvability
Source: PLoS Genet. 2022 Feb 17;18(2):e1010030. doi: 10.1371/journal.pgen.1010030 (PMC8890733; doi:10.1371/journal.pgen.1010030)
Supplement: S2 Table — Asterisks indicate phosphorothioated bond and underlined letters indicate the mutated bases. (DOCX) [file pgen.1010030.s008.docx]

| mutation | background | Oligo sequence |
| --- | --- | --- |
| A3321T  (K1107N) | 7-mut | C*A*T*C*GCTGGCAAACGTATACGGCGGAATATTTGCCGAATACCGTGTGGACGTAAGCGTGAACGTCAGGATCACGTTTCCCCGACCCGCTG |
| A2959G  (T987A) | 6-mut and 7-mut | C*G*C*C*ACCTTTACAATGTCCCCGACGATTTTTTCCGCCCTCAGCGCACCGTTTATCGTACAGTTTTCAGCTATCGTCACATTACTGAGCG |
| T3364C  (F1122L) | 6-mut and 7-mut | T*C*T*G*TAACACACTCAGACCACGCTGATGCCCAGCGCCTGTTTCTTAAGCACCATAACCTGCACATCGCTGGCAAACGTATACGGCGGAAT |
| A2959T  (T987S) | 6-mut | C*G*C*C*ACCTTTACAATGTCCCCGACGATTTTTTCCGCCCTCAGCGAACCGTTTATCGTACAGTTTTCAGCTATCGTCACATTACTGAGCG |
| C2909A  (S970Y) | 6-mut, 6-mut T987A, and 7-mut | T*T*A*T*CGTACAGTTTTCAGCTATCGTCACATTACTGAGCGTCCCGTAGTTCGCATTCACACTGCCACTGATATCCGCATTTTTAGCGGTCA |
| S987L  (C2960T) | 6-mut T987S | C*G*C*C*ACCTTTACAATGTCCCCGACGATTTTTTCCGCCCTCAGCAAACCGTTTATCGTACAGTTTTCAGCTATCGTCACATTACTGAGCGT |
| S1011R  (C3033G) | 6-mut S987L | A*T*C*G*GTCACGGTGACAGTACGGGTACCTGACGGCCAGTCCACACCCCTTTCACGCTGGCGCGGAAAAGCCGCGCTCGCCACCTTTACAAT |
| S1049R  (C3147A) | 6-mut | G*T*T*C*ATCAGTACTTTCAGATAACACATCGAATACGTTGTCCTGCCTCTGACAGTACGCTTACTTCCGCGAAACGTCAGCGGAAGCACCAC |
| galK t435a  (premature stop codon) | Any CI857 derivative | G*C*T*T*CACTGGAAGTCGCGGTCGGAACCGTATTGCAGCAGCTTTAACATCTGCCGCTGGACGGCGCACAAATCGCGCTTAACGGTCAGGAA |
| Codon 987 library | 6-mut | C*G*C*C*ACCTTTACAATGTCCCCGACGATTTTTTCCGCCCTCAGNNNACCGTTTATCGTACAGTTTTCAGCTATCGTCACATTACTGAGCG |
