# Supplementary material for: Viral protein instability enhances host-range evolvability
Source: PLoS Genet. 2022 Feb 17;18(2):e1010030. doi: 10.1371/journal.pgen.1010030 (PMC8890733; doi:10.1371/journal.pgen.1010030)
Supplement: S3 Table — (DOCX) [file pgen.1010030.s009.docx]

| genotype | date collected | unadjusted decay rate | adjusted decay rate |
| --- | --- | --- | --- |
| 6-mut A | 63021 | -0.567 |  |
| 6-mut B | 63021 | -0.554 |  |
| 6-mut C | 63021 | -0.542 |  |
| ancestral 𝞴 A | 63021 | -0.145 | 0.262 |
| ancestral 𝞴 B | 63021 | -0.123 | 0.222 |
| ancestral 𝞴 C | 63021 | -0.228 | 0.411 |
| 6-mut A | 82420 | -0.575 |  |
| 6-mut B | 82420 | -0.541 |  |
| 6-mut C | 82420 | -0.543 |  |
| 7-mut A | 82420 | -0.582 | 1.053 |
| 7-mut B | 82420 | -0.547 | 0.989 |
| 7-mut C | 82420 | -0.484 | 0.875 |
| 6-mut T987A A | 82420 | -0.163 | 0.295 |
| 6-mut T987A B | 82420 | -0.124 | 0.224 |
| 6-mut T987A C | 82420 | -0.130 | 0.235 |
| 6-mut F1122L A | 82420 | -0.117 | 0.212 |
| 6-mut F1122L B | 82420 | -0.163 | 0.295 |
| 6-mut F1122L C | 82420 | -0.112 | 0.202 |
| 6-mut | 60521 | -0.541 |  |
| 6-mut | 60521 | -0.544 |  |
| 6-mut | 60521 | -0.537 |  |
| T987S | 60521 | -0.123 | 0.228 |
| T987S | 60521 | -0.168 | 0.311 |
| T987S | 60521 | -0.161 | 0.298 |
| T987C | 60521 | -0.211 | 0.390 |
| T987C | 60521 | -0.215 | 0.398 |
| T987C | 60521 | -0.220 | 0.407 |
| T987Y | 60521 | -0.111 | 0.205 |
| T987Y | 60521 | -0.157 | 0.291 |
| T987Y | 60521 | -0.134 | 0.247 |
| T987R | 60521 | -0.155 | 0.287 |
| T987R | 60521 | -0.138 | 0.255 |
| T987R | 60521 | -0.119 | 0.220 |
| 6-mut | 21821 | -0.487 |  |
| 6-mut | 21821 | -0.446 |  |
| 6-mut | 21821 | -0.492 |  |
| T987L | 21821 | -0.485 | 1.022 |
| T987L | 21821 | -0.470 | 0.989 |
| T987L | 21821 | -0.511 | 1.075 |
| T987G | 21821 | -0.160 | 0.336 |
| T987G | 21821 | -0.103 | 0.218 |
| T987G | 21821 | -0.153 | 0.321 |
| 6-mut | 33121 | -0.502 |  |
| 6-mut | 33121 | -0.482 |  |
| 6-mut | 33121 | -0.482 |  |
| T987K | 33121 | -0.211 | 0.433 |
| T987K | 33121 | -0.085 | 0.174 |
| T987K | 33121 | -0.108 | 0.222 |
