# Supplementary material for: Viral protein instability enhances host-range evolvability
Source: PLoS Genet. 2022 Feb 17;18(2):e1010030. doi: 10.1371/journal.pgen.1010030 (PMC8890733; doi:10.1371/journal.pgen.1010030)
Supplement: S4 Table — Positive growth rates are bold. Growth rates could not be calculated (“NA”) for some populations (6-mut T987A 2 and 6-mut F1122L 1 and 2) because no viable phage were detected at day 6 (i.e. the population had gone extinct), or because phage decayed to zero during the overnight growth period on lamB— (6-mut T987S 4). Growth rates for each population were measured in a single replicate. (DOCX) [file pgen.1010030.s010.docx]

| starting genotype | population | growth rate |
| --- | --- | --- |
| 6-mut | 1 | **0.79** |
| 6-mut | 2 | **0.04** |
| 6-mut | 3 | **0.66** |
| 6-mut | 4 | **0.60** |
| 6-mut | 5 | **0.86** |
| 6-mut | 6 | **1.38** |
|  |  |  |
| 6-mut T987A | 1 | -0.04 |
| 6-mut T987A | 2 | NA |
| 6-mut T987A | 3 | **0.95** |
| 6-mut T987A | 4 | -0.08 |
| 6-mut T987A | 5 | -0.18 |
| 6-mut T987A | 6 | -0.03 |
|  |  |  |
| 6-mut T987S | 1 | **0.09** |
| 6-mut T987S | 2 | -0.15 |
| 6-mut T987S | 3 | -0.16 |
| 6-mut T987S | 4 | NA |
| 6-mut T987S | 5 | **0.04** |
| 6-mut T987S | 6 | -0.03 |
|  |  |  |
| 6-mut F1122L | 1 | NA |
| 6-mut F1122L | 2 | NA |
| 6-mut F1122L | 3 | -0.06 |
| 6-mut F1122L | 4 | -0.09 |
| 6-mut F1122L | 5 | -0.10 |
| 6-mut F1122L | 6 | -0.07 |
