# Supplementary material for: Viral protein instability enhances host-range evolvability
Source: PLoS Genet. 2022 Feb 17;18(2):e1010030. doi: 10.1371/journal.pgen.1010030 (PMC8890733; doi:10.1371/journal.pgen.1010030)
Supplement: S1 Text — First, a description of a high throughput genetic engineering method and assay for evaluating whether lambda strains are one mutation (N1107K) away from gaining the use of OmpF. Second, a description of a more sensitive assay for detecting OmpF use than plate-based spot tests. (DOCX) [file pgen.1010030.s011.docx]

**S1 Text**

**High throughput Cos-MAGE to edit N1107K into engineered backgrounds**

Initially, we edited N1107K into only a subset of all variant backgrounds. To do this, we used a low throughput method of Cos-MAGE, requiring isolation and sequencing individual lysogen clones and inducing phage production. We later sought to confirm the effect of N1107K in all backgrounds, so we designed a modified Cos-MAGE procedure to increase throughput and reduce cost of searching for clones that received the mutation. To do this, we conducted two cycles of CoS-MAGE in each remaining background using the appropriate galK oligo as well as the N1107K oligo, with three replicates per background. Following an overnight recovery step, cultures were induced using the same procedure as described in the section **Induction of lysogens by heat shock.** Lysates were filtered, diluted in LBM9, and spotted on two different lawns: one containing WT cells and the other containing *lamB*^—^ cells. The titer on WT lawns provided the total number of phage particles in the lysate, and the titer on *lamB*^—^ cells provided the number of phage that had been converted to OmpF^+^ after MAGE with the N1107K oligo. We included 6-mut as a positive control and observed a 10-20% conversion rate. As an additional control to verify that the MAGE process was working in each variant, we computed the conversion rate of the *galK* selectable marker as a positive control and observed a conversion rate of 3-12%. Given the high density of lysogen cells prior to induction (>10^7^) it is unlikely that our method failed to detect conversions to OmpF^+^. In S1 Table, we present the conversion rates both of the *galK* selectable marker and of the phage genotypes to OmpF^+^. When no OmpF^+^ conversions were detected, we calculated an upper bound for the conversion rate if a single plaque had been detected.

**Liquid assay to detect weak growth on OmpF after editing in N1107K**

Upon receiving the N1107K mutation, most backgrounds either produced obvious plaques when serial dilutions were spotted on *lamB*^—^ lawns (6-mut, T987L, and T987C) or produced no visible effect when spotted on *lamB*^—^ (F1122L, T987S, T987G, T987K, and T987R). However, two backgrounds, T987A and T987Y, failed to produce individual plaques in a dilution series, but produced turbid clearings when high concentrations of phage were spotted. We hypothesized that perhaps these two backgrounds gained the ability to infect using OmpF at very low levels, resulting in some killing of cells but at a rate too low to produce plaques. To test this, we designed an assay to more sensitively measure growth rate on *lamB*^—^ cells. For this assay we chose representative variants that produced good plaques (6-mut + N1107K), no plaques but turbid clearings (T987A + N1107K), and no visible effect (F1122L + N1107K) and inoculated three replicate tubes with 10 $\mu L$ of filtered lysate and 100 $\mu L$ of *lamB*^—^ cells to 4 mL LBM9 supplemented with 40 $\mu L$MgSO_4_ and incubated at 37 °C shaking for 14.5 hours. Phage growth was quantified by plating on the permissive host (WT) before and after phage were incubated with *lamB*^—^ cells. We quantified pre-growth densities by diluting phage in LBM9 (before cells were added) and spotting 2 $\mu L$ of each dilution a WT lawn. Post-growth densities of phage were obtained by chloroforming cultures (to remove cells) and diluting phage in LBM9 and spotting 2 $\mu L$ of each dilution on a WT lawn. We found that, as predicted, genotypes that produce no visible effect on plates do not measurably grow on *lamB*^—^ in liquid culture, whereas genotypes that produce turbid clearings grow at a measurable but significantly reduced rate compared to genotypes that plaque well on *lamB*^—^ lawns (S4 Fig). We compared growth rates among genotypes using paired t-tests corrected for multiple comparisons using the Bonferroni method.
