# Supplementary material for: Viral protein instability enhances host-range evolvability
Source: PLoS Genet. 2022 Feb 17;18(2):e1010030. doi: 10.1371/journal.pgen.1010030 (PMC8890733; doi:10.1371/journal.pgen.1010030)
Supplement: Attachment — Submitted filename: strobel_plos_response_to_reviewers.docx [file pgen.1010030.s012.docx]

**Reviewer #1: In this paper, Strobel et al. attempt to examine the link between thermostability and evolvability. They use bacteriophage lambda which they have previously shown can evolve from using only LamB as a receptor to also being able to use OmpF as a receptor. Previously, they found that an isolate with 7 mutations (7-mut) had the ability to use LamB and OmpF but was much less stable than the ancestor. They reversed a key mutation for OmpF use and found that the resulting isolate (6-mut) was still unstable. They then introduce two different stabilizing mutations to 6-mut. Following experimental evolution, all populations of 6-mut rapidly gained the ability to use OmpF whereas the stabilized populations took much longer, and many populations did not evolve the ability or went extinct. Sequencing revealed individual mutations gave 6-mut the ability to grow on OmpF but each stabilized population required 2 separate mutations to evolve to grow on OmpF. Next, they mutate one of the residues which increased thermotolerance to generate a panel of mutants. These mutants differed in thermostability and following experimental evolution, the more thermostable mutants evolved slower with a single exception (T987C). Again, thermostable isolates required 2 mutations.**

**Next, the authors use Alphafold to model the predicted structure of the viral protein J, the host recognition protein, to show the predicted location of the mutations from their experiments. This showed that mutations allowing the use of OmpF were in sensible places on an exposed surface of the protein. They then show that the double mutations required for the thermostable variants to evolve to use OmpF, the first mutation destabilizes the phage before the second allows the use of OmpF. Introducing, only the mutation allowing the use of OmpF to thermostable isolates did not allow robust use of OmpF without an additional mutation. Finally, they measure growth rates of the variants and show that T987C has significantly lower growth compared to all the other isolates which explains why it might not evolve naturally. The authors propose a model that J must be destabilized such that it can access alternative forms of the protein which can bind to OmpF.**

**The paper is clearly written, and the experiments are easy to follow. The authors have generated some interesting data and this paper forms part of an important debate on the relationship between thermostability and evolvability. I especially like the model that the authors propose that viral attachment proteins must be unstable to infect a broader range of hosts. However, I think the authors should address 3 major things before publication.**

**We would like to thank the reviewer for their thoughtful summary and helpful suggestions. We will address specific comments below.**

**1. The authors don’t discuss the limitation of their experiment with 6-mut being a single mutation away and 1122 being effectively a reversion of a previous mutation.**

To address this concern raised by both reviewers, we conducted an additional experiment to test the effect of the T987A stabilizing mutation in a background that is further away from the 7-mut. To do this, we reverted another of the 7-mut mutations to create a 5-mut, and then engineered in the T987A stabilizing mutation, confirmed that it is more stable than the 5-mut, and tested whether it is more or less evolvable than 5-mut. We found that, in line with our model, 5-mut T987A is less evolvable than 5-mut, and the difference in evolvability was even more pronounced than the difference between 6-mut and 6-mut T987A. This result demonstrates that the initial results were not a special case unique to the 6-mut genotype. These new results are reported in the last paragraph of the results and Fig. 7.

Also, the F1122L stabilizing mutation is not a reversion of a 6-mut mutation. This is explained further in a later response.

**2. The modelling section needs more discussion of the model generated and the limitations of this approach.**

We have addressed this concern with specific revisions discussed below.

**3. I would like more discussion of the limitations of the experimental design and more links to the published literature.**

We have added additional experiments and paragraphs in the manuscript to address this concern, and we address specific points related to this comment in much greater detail below.

**Major Issues**

**1. I have a conceptual issue with the paper. The authors start with an isolate which is a single mutation away from 7-mut, the isolate with ability to use OmpF. Unsurprisingly, OmpF use can evolve very quickly as there were a large number of phage in the initial population. This was compared to isolates with additional stabilizing mutations.**

We carefully selected our initial population size to ensure that OmpF^+^ phage evolved during the evolution experiment, rather than being present at low frequency in the initial inoculum. Each flask was seeded with an inoculum of 10^6^ phage, between 1/10th and 1/100 of the size that would be required to sample all single mutations (at least 10^8^, given that the published per base mutation rate for bacteriophage lambda is 7.7 x 10^-8^). Additionally, when initiating the experiment, we plated more than equivalent volume used to start the experiment on L- lawns, and did not observe any plaques. Further, we do not rely on time as our only measure of evolvability. We show that stabilized genotypes have a longer mutational pathway to traverse on the way to OmpF^+^ compared to unstable genotypes.

**However, the authors in their previous work have already shown that the key mutations allowing the use of OmpF are destabilizing. When tracking the mutations, the stabilizing mutation reverted before the OmpF allowing mutation arose. In fact, 1122 was the site of one of the mutations in 6-mut so using it as a thermostability mutation is in effect creating a 5-mut. It is not then surprising that the thermostable mutants took longer to evolve on OmpF.**

In the ancestor, the amino acid at 1122 is I (isoleucine), and it becomes F (phenylalanine) in the 6-mut. The stabilizing mutation is F1122L (leucine). Isoleucine and leucine are chemically similar, but this does not necessarily mean that both amino acids behave the same in the protein structure, and so there is no reason to expect that the F1122L stabilized 6-mut would be equivalent to a 5-mut. That being said, we recognized and explicitly noted the potential confounding effects associated with the 1122 position in the manuscript, and that was why we designed the library of variants at position 987, a site that had not been previously mutated.

**Is this therefore a good/fair test of evolvability? Is 1122, the key mutation that destabilizes 6-mut?**

Petrie *et al*., 2018 (cited in manuscript), the source of the 7-mut strain, generated a genotype with some of the same amino acid substitutions as 7-mut, (1012G and 1107K), but lacking the mutation at 1122, and that genotype is intermediately unstable between the ancestor and 7-mut. This suggests that 1122 alone is not responsible for destabilizing 6-mut or 7-mut.

**The authors are in effect taking something where it is known that the required mutations are destabilizing, reversing those mutations and showing that it takes longer to evolve the trait.**

Although prior work showed that the OmpF^+^ was less stable than the OmpF^-^ ancestor, the specific mutation we chose to revert in the 7-mut was not a destabilizing mutation. In this study, we reverted N1107K in the 7-mut and showed that there was no change in stability. We then identified two naturally selected stabilizing mutations, one of which occurred at a site that was previously mutated in the 7-mut, but it was *not* a reversion. The other stabilizing mutation occurred at a site (987) that was not mutated in the 7-mut, and was distant from any of the 7-mut mutations, both in the amino acid sequence and in the predicted 3-dimensional structure.

**There was no way that anything could evolve faster than 6-mut as it required just a single mutation which happened within a passage or two.**

It is true that nothing could evolve faster than 6-mut. Had the stabilized genotypes evolved in a single mutation like the unstable genotypes, we would not have been able to draw conclusions about the effects of the stabilizing mutations on evolvability. However, the result we obtained was that most stabilizing mutations actually lead to slower evolution, requiring longer mutational pathways. As to whether increasing the mutational pathway length from 1-mutation to 2-mutations is trivial, our results suggest that it is not. Doubling the mutational pathway length caused much greater than doubling the number of days required to evolve OmpF^+^.

**It seems likely that for epistatic reasons, a more stable J cannot accept the OmpF-using mutations and therefore required 2 mutations.**

We found that the OmpF-using mutations generate different phenotypes (OmpF^-^ vs. OmpF^+^) depending on the background in which they appear (stable vs. unstable). We do not disagree that this is an example of epistasis. It is important to note, though, that this epistasis occurs *only* with respect to OmpF use, but not with use of the ancestral receptor, LamB. This suggests that the epistasis between background and stabilizing mutations is not a generic incompatibility between mutations, but reflects an incompatibility between high stability and high evolvability.

**All this being said, even if the experimental design is not perfect, I still believe that this data is significant, and it highlights that for the phage to switch receptors, it requires a destabilization of the protein. I think the authors should discuss whether this is a special case of evolvability and discuss the caveats of their approach in much greater detail.**

We have added a new experiment (Fig. 7) to respond to the critique that we only studied one genotype. We also have added an additional paragraph discussing whether our findings are applicable to evolution in nature and sentences in our final paragraph adding caution to the applicability of our results to the evolution of animal viruses.

**2. The Alphafold modelling section needs to be reworked. Firstly, Alphafold provides a confidence in its own model prediction. This data needs to be shown so that we can see whether the conclusions drawn from the model are likely. I would guess that the 960-990 region is low confidence which would not be surprising because it could likely interact with the N-terminal portion of the protein. The decision to model only 173 amino acids rather than the full protein should be discussed not just in the methods. Maybe a schematic should be provided of the known domains of J. Alphafold models exist of longer proteins than J so the authors could try to model the full structure. The accuracy of the Alphafold prediction is greatly improved by having a good Multiple Sequence Alignment (MSA) of related proteins. What was the depth of the MSA, how did this affect the confidence in the prediction? Are there any related structures which have been experimentally determined that this structure could be compared to? Finally, the authors could consider trying to model how their structure might interact with LamB or OmpF although this may be beyond the scope of the paper.**

We encountered computational limits using the Google Alphafold colab notebook to predict the full 1133 amino acid structure, and it is stated on the website that memory and space are known issues with predicting long structures.

We have added the following sentences to the main text to discuss more why we chose the shorter sequence to model:

“We modelled the protein multiple times by varying which segments of the protein to include. In the end, we found that the segment with the highest confidence score and for which all the mutations being studied were included, was a 173 amino acid segment at the C-terminal end. The problem with predicting a larger portion of the protein is that from ~amino acid 780 to 960, the model confidence is very low (40-60%) compared to the terminal region (80-90%). The J region we chose is known through biochemical assays to be the portion that binds the receptor [*28*], it encompasses the majority of host-range altering mutations previously reported [*25, 27*], and has been shown to have an elevated rate of evolution in nature.”

We have also modified Fig. S2 to show the sequence coverage and model confidence for each model (ancestor and 6-mut) for the region that we modelled (960-1132). The 6-mut model is the same as in Fig. 4. For most of the modelled sequence, coverage is >50, which is well above the threshold set by Alphafold of 30 sequences. The model confidence is also high (>75/100) for most of the sequence.

We were very excited to be able to include and discuss the Alphafold prediction in this manuscript. However, it is not the main focus of the manuscript, and we believe that additional work related to modelling the J protein, such as domain analysis and receptor binding, is beyond the scope of the current manuscript. We are very excited to be currently working on additional experimental and computational work on the J protein for future publication.

**3. I would like to see more discussion especially of the limitations of the paper and comparing to other research. For example, mutations at a single site are unlikely to have independent effects from each other but the authors don’t mention this as a pretty big limitation of their experimental design.**

We see the validity in the concern about non-independence. However, given that much of the experimental work to date on the stability-evolvability debate has hinged on comparisons between viruses or proteins differing at many sites, we also see value in comparing genotypes that are identical except for a single site. We added a sentence to the manuscript to this effect:

“That our library variants were genetically identical except for the amino acid at a single site was both a strength and a limitation of our study. This design allowed us to pinpoint and precisely manipulate the genetic basis for stability and evolvability in λ; however, focusing in on a single site could result in non-independent effects.”

**What are the effects of performing this evolution experiment in laboratory conditions? The authors by passaging regularly, are removing any cost of a lack of environmental survival. Does a lab environment allow unstable phage that the real world does not? This should be discussed.**

All experiments performed in a laboratory will carry the caveat that they cannot fully represent natural conditions. However, we do have some insights into whether the phage experience a cost for instability in this experiment. We passaged every 24 hours, a timeframe that is likely much longer than required for the bacteria to reach carrying capacity and stop growing exponentially. Once bacteria stop growing, phage will no longer be able to replicate at high rates and will have to survive in the flask environment until the next transfer gives the bacteria access to fresh media and triggers them to begin growing exponentially again. This time period imposes selection for survival. Data from our manuscript provide insight into the intensity of this selection: the decay rates we reported in the manuscript were measured at the same temperature as the evolution experiment (37 C), and even for the stable genotypes, they are losing ~60% of their population every 6 hours to decay (for unstable genotypes it’s >90%). This indicates that phage are constantly dying in these environmental conditions and there is selection for survival.

Additionally, prior work on this phage has demonstrated that the same sites that evolve mutations in laboratory conditions are also hotspots for molecular evolution in natural populations. We have added a paragraph discussing this unique benefit of our phage.

**I think that the authors should engage more with the literature about why their results differ from the consensus. I think the current model suggests that evolvability can be increased by having a stable core which allows an active site to change without destroying the protein fold. This paradigm has mostly been applied to globular enzymes. This paradigm might not apply to bacteriophage attachment proteins. This paper suggests to me that to evolve to use a new receptor, J must be more flexible which in turn reduces the stability of the phage.**

We have added the following sentences to the discussion to further contextualize our results.

“Additionally, prior work on this question has focused primarily on enzyme proteins, and it is not entirely surprising that the paradigm that emerged from those studies might not apply to viral attachment proteins, especially if viral proteins generally have different properties from cellular proteins.”

**There is research on bacteriophage life history trade-offs that discusses this hypothesis. More examples of different viruses evolving would also benefit the discussion.**

Although the literature is replete with examples of virus host-shifts, very few actually address the key question of interest in this study, which is how does stability affect host-shift evolvability. To keep the literature review focused, we decided to highlight only the most relevant to this study.

**Minor Issues**

**1. Figure 3A should be presented the other way up. It’s easier if there is consistency between the figures so higher values = more stable.**

Reporting the relative decay rates in Fig. 3A stemmed from our intention to be fully transparent with the data, since we did detect mild differences between replicates of the same genotype measured on different days. Upon receiving the reviewer’s comment, we do see how reporting the relative decay rates is confusing, since the axes are flipped relative to Fig. 1. On closer inspection of the day-effects, we realized that since we measured each variant alongside 6-mut, we could perform statistical comparisons to 6-mut without performing the adjustment. For comparisons to the $\lambda$ancestor, we performed the statistics both ways (with raw decay rates and with relative decay rates) and discovered that the choice did not affect the outcome of any comparison. Therefore we chose to graph and report statistics on the raw decay rates, but to provide a table with both the raw and relative decay rates (Table S3).

|  | Bonferroni corrected on RAW rates | Bonferroni corrected on RELATIVE rates |
| --- | --- | --- |
| ancestor 𝝀 - 7-mut | * | * |
| ancestor 𝝀 - 6-mut T987A | ns | ns |
| ancestor 𝝀 - 6-mut F1122L | ns | ns |
| ancestor 𝝀 - 6-mut T987L | * | * |
| ancestor 𝝀 - 6-mut T987C | ns | ns |
| ancestor 𝝀 - 6-mut T987S | ns | ns |
| ancestor 𝝀 - 6-mut T987G | ns | ns |
| ancestor 𝝀 - 6-mut T987K | ns | ns |
| ancestor 𝝀 - 6-mut T987R | ns | ns |
| ancestor 𝝀 - 6-mut T987Y | ns | ns |
|  |  |  |

**2. Please add Figure 6 stats.**

Figure 6 stats have been added. Measurements in figure 6 were taken across two days, and in the initial submission we separated genotypes measured on different days and performed statistical tests on the separated datasets, even though there were no detectable day-effects. In the revised version, we decided that the results would be clearer if we report statistics on the combined datasets, especially since day-effects were not a problem. The outcomes of some comparisons were slightly affected (6-mut vs. 6-mut F1122L became significant and 6-mut vs. 6-mut T987G became marginally significant), but these changes did not affect our main conclusions because most growth rates remained on par with 6-mut or higher. This is further explained in a response below.

**3. L40. I think the authors can assert that viruses are highly evolvable.**

We have edited this sentence to reflect the comment.

**4. L59. 2019 rather than 2020 as the start of the pandemic.**

We have edited this sentence to reflect the comment.

**5. L88 non-native conformers. An additional sentence explaining what this means would be helpful as I was not familiar with this phrase.**

We have added a definition to that sentence:

Indeed, the conformational flexibility of unstable proteins can be essential for evolvability by allowing non-native conformers, protein molecules that fold into conformations other than the ground-state conformation, to explore promiscuous functions [*18, 19*].

**6. L94. Did the authors mean apposing here?**

We have corrected the sentence.

**7. L138 and elsewhere. The authors perform multiple T tests on their data where I think that a 1-way ANOVA correcting for multiple comparisons would be more appropriate and is what the authors use later.**

The Bonferroni correction is used when there are a planned number of comparisons ahead of time, whereas the Tukey HSD is intended for comparing all possible combinations or when comparisons were not pre-planned. Since our purposes fall into the former category, we selected the Bonferroni correction. We knew which comparisons we were interested in ahead of time and never intended to compare all possible pairs. The Bonferroni correction is a widely used and accepted method (including in recently published PLoS Genetics papers) and tends to be more conservative than the Tukey post-hoc test. For consistency, we now report Bonferroni corrected p-values whenever appropriate in the paper rather than Tukey post hoc p values. In order to fully consider the point being made by the reviewer, we also conducted ANOVA and post-hoc Dunnett tests (more appropriate for most of our analyses, which were many-to-one comparisons of our variant genotypes to 6-mut), or post-hoc Tukey tests in the case of the destabilizing mutation dataset (which was not a many-to-one scenario). In no case did the outcome of the comparison (significant vs. nonsignificant) depend on which method was chosen (post hoc Tukey/Dunnet vs. paired t-tests with Bonferroni correction). Since the Bonferroni method was more conservative in all cases, we chose to report those p values in the paper.

We present the results of that test here:

| DECAY RATES |  |  |
| --- | --- | --- |
|  | t-test bonferroni corrected | Dunnett's post hoc |
| 6-mut - ancestor 𝝀 | * | **** |
| 6-mut - 7-mut | ns | ns |
| 6-mut - 6-mut T987A | *** | **** |
| 6-mut - 6-mut F1122L | ** | **** |
| 6-mut - 6-mut T987L | ns | ns |
| 6-mut - 6-mut T987C | **** | **** |
| 6-mut - 6-mut T987S | *** | **** |
| 6-mut - 6-mut T987G | ** | **** |
| 6-mut - 6-mut T987K | * | **** |
| 6-mut - 6-mut T987R | *** | **** |
| 6-mut - 6-mut T987Y | *** | **** |
|  |  |  |
|  | t-test bonferroni corrected | Dunnett's post hoc |
| ancestor 𝝀 - 7-mut | * | **** |
| ancestor 𝝀 - 6-mut T987A | ns | ns |
| ancestor 𝝀 - 6-mut F1122L | ns | ns |
| ancestor 𝝀 - 6-mut T987L | * | **** |
| ancestor 𝝀 - 6-mut T987C | ns | ns |
| ancestor 𝝀 - 6-mut T987S | ns | ns |
| ancestor 𝝀 - 6-mut T987G | ns | ns |
| ancestor 𝝀 - 6-mut T987K | ns | ns |
| ancestor 𝝀 - 6-mut T987R | ns | ns |
| ancestor 𝝀 - 6-mut T987Y | ns | ns |
|  |  |  |
| DESTABILIZING MUTATIONS |  |  |
|  | bonferroni adjusted sig | Tukey post hoc |
| 6-mtu vs. 6-mut T987A | ** | **** |
| 6 -mut T987A vs. 6-mut T987A S970Y | * | ** |
| 6-mut T987A S970Y vs. 6-mut T987A S970Y N1107K | ns | ns |
|  |  |  |
| 6-mut vs 6-mut F1122L | *** | **** |
| 6-mut F1122L vs. 6-mut F1122L L122F | *** | **** |
| 6-mut F1122L L122F vs. 6-mut F1122L L1122F S1049R | ns | ns |
|  |  |  |
|  |  |  |
| 6-mut vs. 6-mut T987S | *** | **** |
| 6-mut T987S vs. 6-mut T987S T987L | *** | **** |
| 6-mut T987S T987L vs. 6-mut T987S T987L S1011R | ns | ns |
|  |  |  |
|  |  |  |
|  |  |  |
| GROWTH RATES |  |  |
|  | Bonferroni corrected | Dunnett's post hoc |
| ALA-sixmut | * | * |
| ARG-sixmut | ns | ns |
| CYS-sixmut | ** | *** |
| FelevenL-sixmut | *** | *** |
| GLY-sixmut | * | * |
| LEU-sixmut | ns | ns |
| LYS-sixmut | ns | ns |
| SER-sixmut | ns | ns |
| TYR-sixmut | ns | ns |

**8. L152- State that the coevolved host is LamB-.**

We added the following sentence to clarify:

This time-shifted host had a duplication in the *malT* gene, a regulator of LamB expression, causing LamB to only be expressed in rare mutants that had reverted the duplication (41).

**9. L173-L175. Should state that L1122F is a direct reversion. These sentences could be better integrated into the paragraph. Rephrase to make a little clearer.**

To clarify, L1122F is a direct reversion of the stabilizing mutation, not of one of the 6-mut mutations. In the ancestor, the amino acid at 1122 is I, and it mutates to F in the 6-mut. The stabilizing mutation that occurred in the 6-mut background is F1122L. In one of the coevolution populations initiated from the 6-mut F1122L, we observed a genotype that had a mutation L1122F, which was a direct reversion of the stabilizing mutation.

**10. L204- No need to give the base changes. Amino acid changes only would be clearer.**

We have edited this sentence to reflect the comment.

**11. Supplemental figure S3. This figure is not supplemental in my opinion but is key to understanding the entire mechanism proposed. This data demonstrates that N1107K does not give the same effect in a stabilized J protein which is why 2 mutations are required. I think it (or a quantification of the results) should be moved to a main figure.**

In our opinion the results of the evolution experiments are much more central to the paper, since Fig S3 involves engineering in a single mutation whereas the evolution experiments capture all possible pathways taken by genotypes. We already discuss the results of Fig S3 extensively in the main text, but we disagree that the plate photo or the quantitation shown in (Fig. S 4B) should be a main figure.

**12. L310-311 Not clear when and where the authors expected this mutation to evolve.**

T987C was the only stable variant that evolved OmpF^+^ with a single mutation on the surface but no additional mutation. Our model would predict that T987C should have required another mutation beyond a surface mutation in order to use OmpF. However, this paragraph describes how a path could exist for T987C to evolve without the additional mutation.

**13. L317 Kruskal Wallis or ANOVA? Which one? Why was Kruskal Wallis used later on but ANOVA before?**

Initially, we separated the growth rate dataset into groups parts and analyzed them separately. The reason for this was that most genotypes were measured during a single experiment, but F1122L and T987C were not included in that first experiment so we measured their growth rates on a different day, alongside three additional replicates of 6-mut for comparison. When considered separately, there were some pairs of measurements that violated the assumption of homogeneity of variance, and therefore we analyzed the data with a Kruskal-Wallis test.

However, upon further consideration prompted by the reviewer’s comment, we sought ways to simplify our analysis. We tested whether there were any day effects between the 6-mut replicates measured during the two separate experiments, and there were no differences, so we decided to combine the two datasets and analyze the results together. The combined dataset did not violate the homogeneity of variance assumption, so we more statistically powerful parametric tests. This new analysis did not substantively change the results, except that the increased power revealed a significant difference in the F1122L growth rate relative to the 6-mut and a marginally significant difference for 6-mut vs. 6-mut T987G. We have revised the sections on growth rate accordingly.

**14. L410-414. This is quite speculative. I can see links between this work and the propensity for furin cleavage in S and up/down conformations. The authors could discuss this in more detail though not if they don’t want to. Given how variants have outcompeted the initial strain, I don’t know how great an example this is for the paper even if topical.**

It is fully consistent with our model that the initial, unstable strain that first jumped into humans would be outcompeted later by descendant strains that specialize on growth in the new host (humans). Past work from our lab has shown that after an unstable lambda first evolves to use OmpF, specialists on OmpF evolve and regain stability (J. R. Meyer, D. T. Dobias, S. J. Medina, L. Servilio, A. Gupta, R. E. Lenski, Ecological speciation of bacteriophage lambda in allopatry and sympatry. *Science*. 354, 1301-1304 (2016).

**-----------------------------------------------------------------------------------------------------------------------------**

**Reviewer #2: I understand that the authors want to publicize their work by referring to viral emergence and the COVID pandemics. However, this article deals with a very specific phage-bacterial system, and extrapolations to the use of receptors in animal viruses are not warranted in this case. Therefore, I suggest refocusing these references (last paragraph, author's abstract). Host shifts are also relevant in the bacterial world, for example for phage therapy. I suggest that the word "emergence" be removed from the title.**

We have changed the word “emergence” to “host-range” in the title and altered the abstract, author summary, and to more generally discuss virus host-range evolution. However, we would like to push back on the idea that bacteriophages provide no useful insights into animal virus evolution. Leaders in the field of experimental evolution routinely use bacteriophages as model systems to broadly understand the evolution of viruses, and previous papers have discussed how their results provide insights into the evolution of medically relevant viruses (two highly cited examples are listed below). With that in mind, we added a sentence in our final paragraph to make our conclusions more conservative with respect to animal virus applications.

“Our results were derived from a study of bacteria-infecting virus rather than an animal virus, and further work is warranted to investigate the role of viral particle instability in animal virus host-range evolvability.”

And we also add specific recommendations for how our findings might be applied in this context.

R. C. McBride, C. B. Ogbunugafor, P. E. Turner, Robustness promotes evolvability of thermotolerance in an RNA virus. BMC Evolutionary biology 8: 231 (2008).

C. B. Ogbunugafor, R. C. McBride, P. E. Turner. Predicting virus evolution: the relationship between genetic robustness and evolvability of thermotolerance. *Cold Spring Harbor Symposia on Quantitative Biology LXXIV*, 109-118 (2009).

J. M. Cuevas, A. Moya, R. Sanjuán. A genetic background with low mutational robustness is associated with increased adaptability to a novel host in an RNA virus. *J Evol Biol* 22, 2041-2048 (2009).

**The authors measured overall infectious particle stability (decay assays), not the stability of a given protein. This should be OK since mutants were isogenic, but some caveats apply though.**

We acknowledge the reviewer’s hesitance to accept the particle stability as a proxy for the stability of the J protein. We have several pieces of evidence to support our claim.

The first is that, as the reviewer recognized above, the mutants were isogenic. And additionally, they were engineered using oligos with the specific mutation, so it would be highly surprising if the stabilizing effect of all of the mutants were due to mutations appearing elsewhere in the genomes in parallel, despite no selection for particle stability when the phage are in the lysogenic state.

The second piece of evidence is the subject of a project that is currently underway in the lab, and we anticipate will be published in the next two years. We have successfully purified active J protein from ancestral and OmpF^+^ mutants and found that the levels of soluble expression and melting points of the OmpF^+^ J protein are dramatically lower than the OmpF^-^ ancestor. While the OmpF^+^ J proteins we have studied have a slightly different set of mutations than the unstable OmpF^+^ genotypes studied in this paper, we see this as a promising sign that our measurements on whole phage stability are reflective of differences in J protein stability.

These results are beyond the scope of the current paper, but we are eager to prepare this work for publication soon.

Left: Thermal shift assay showing melting curves for lambda ancestor (purple); Orange: OmpF+ lambda (Orange). The inverse peaks at ~45 C (orange) and ~62 C (purple) show a difference in melting point of >15 C. Right: lane 1: ladder, lane 2: empty, lane 3: ancestor protein unpurified supernatant, lane 4: OmpF+ protein unpurified supernatant, lane 5: ancestor protein purified, lane 6: OmpF+ protein purified. The much lower intensity of the bands in lanes 4 and 6 relative to 3 and 5 are indicative of less soluble expression (potentially indicating lower stability).

**Since the experiments are based on modifying thermostability by changing one or a few sites, the question arises whether the observed differences in evolvability are due to specific epistatic interactions between sites apparently affecting stability and gain-of-function mutations. The authors select a site (987) that is apparently physically separate from the region that determines ompF evolution. However, this does not exclude these possible "confounding effects", as interactions between the sites could occur in any case.**

We do not rule out the possibility that there are specific interactions between the site 987 and the region that determines OmpF evolution, and it is possible (likely even) that stabilizing different regions of the protein would result in different effects on evolvability. As to whether the loss in evolvability is only due to epistasis between stabilizing and gain of function mutations, we address this with additional experiments reported in the manuscript:

“Along similar lines of thinking, perhaps the extra mutation is required to ameliorate a genetic incompatibility between the stabilizing mutations and the mutations found in the contact surface. This could occur if stabilizing mutations and surface mutations are beneficial alone but produce a non-functional protein when they co-occur in the same genotype. To rule out this possibility, we measured productivity of lysogens for the naturally selected thermostable variants (T987A, F1122L) with and without N1107K and found no difference in titer (Fig. S6) indicating that there is not a genetic incompatibility [*33*]. “

**Finally, the relevance and generality of the conclusions is affected by the fact that, in the model system, the evolutionary trajectory (acquisition of ompF) is rather short (one or few mutations). No other phenotype was examined.**

We conducted additional experiments in response to this concern. To test the effect of a stabilizing mutation in a background that is further away from the OmpF^+^ 7-mut, we started with the 6-mut and reverted another mutation, creating a 5-mut. We then edited in the T987A stabilizing mutation, confirmed that it is stabilizing in the 5-mut background, and measured its evolvability relative to 5-mut. We found that, completely in alignment with our model, the stabilizing mutation inhibits evolvability in the 5-mut background. In fact, the difference in evolvability is even greater than that between 6-mut and 6-mut T987A. These results are reported in the last paragraph in the results and Fig 7.
