# Supplementary material for: Viral protein instability enhances host-range evolvability
Source: PLoS Genet. 2022 Feb 17;18(2):e1010030. doi: 10.1371/journal.pgen.1010030 (PMC8890733; doi:10.1371/journal.pgen.1010030)
Supplement: Attachment — Submitted filename: Rereview.docx [file pgen.1010030.s013.docx]

I would like to thank the authors for responding to my comments. The additional experimental evolution study greatly strengthens their conclusions and the paper.

There remain a few things which have not changed which I believe would benefit the paper.

1. I think the authors should need to add a sentence noting/discussing that an I1122F mutation occurred in the construction of the 7-mut and whether this affects their conclusions with regards to F1122L. It is possible that the larger F residue is destabilizing and as such F1122I would slightly stabilize and F1122L would stabilize more. I do not know whether in Petrie et al., the single reversion of F1122I was studied as opposed to the 5-mut which had 2 mutations. In any case, I think that the authors should discuss the fact that multiple mutations occurred at 1122 even if they decide that it doesn’t affect their results.
2. I really think the authors should present figure 4 with a color scheme as in the alphafold database (e.g. as below) showing the model confidence in their predicted structure. This would allow the reader to directly judge the confidence in applying this structure to the mutations of interest. I appreciate the additional information provided in Fig S2 but it’s not easy to directly translate that to the protein structure. It should be easy to put the data from S2 into the chimera model using render by attribute or another method.

3. L172- Please state that L1122F is a direct reversion of the stabilizing mutation.
4. L314-315. *“If such molecular shortcuts are available to the phage, then why did we not observe T987C evolve naturally?”* I still do not understand in which experiment or environment it was expected that T987C would evolve. I understand that it is surprising that T987C was evolvable and thermostable but that this came at a cost to growth. The authors could rephrase to make this clearer.
