# Supplementary material for: Viral protein instability enhances host-range evolvability
Source: PLoS Genet. 2022 Feb 17;18(2):e1010030. doi: 10.1371/journal.pgen.1010030 (PMC8890733; doi:10.1371/journal.pgen.1010030)
Supplement: Attachment — Submitted filename: Strobel_plos_response_to_reviewers_SECOND_response.docx [file pgen.1010030.s014.docx]

**Response to reviewers, second round**

**Our responses for the second round of revisions are indicated in bold blue text below**

REVIEWER 1

I would like to thank the authors for responding to my comments. The additional experimental evolution study greatly strengthens their conclusions and the paper.

There remain a few things which have not changed which I believe would benefit the paper.

1. I think the authors should need to add a sentence noting/discussing that an I1122F mutation occurred in the construction of the 7-mut and whether this affects their conclusions with regards to F1122L. It is possible that the larger F residue is destabilizing and as such F1122I would slightly stabilize and F1122L would stabilize more. I do not know whether in Petrie et al., the single reversion of F1122I was studied as opposed to the 5-mut which had 2 mutations. In any case, I think that the authors should discuss the fact that multiple mutations occurred at 1122 even if they decide that it doesn’t affect their results.

**We have now added text to explicitly state that 1122 was already mutated in the 7-mut.**

**The possibility that F1122I could be stabilizing but to a lesser extent than F1122L is an intriguing one. The single reversion of F122I was not studied in Petrie et al. nor elsewhere, so we do not know its independent effect on stability. We are wary of speculating based on amino acid structure alone, given that two amino acids with very similar sizes and chemical compositions (threonine and serine) yielded strikingly different phenotypes in our library.**

2. I really think the authors should present figure 4 with a color scheme as in the alphafold database (e.g. as below) showing the model confidence in their predicted structure. This would allow the reader to directly judge the confidence in applying this structure to the mutations of interest. I appreciate the additional information provided in Fig S2 but it’s not easy to directly translate that to the protein structure. It should be easy to put the data from S2 into the chimera model using render by attribute or another method.

**We have updated figure 4 such that the backbone is color coded by model confidence.**

3. L172- Please state that L1122F is a direct reversion of the stabilizing mutation.

**We have now added text to state that L1122F is a direct reversion of the stabilizing mutation.**

4. L314-315. *“If such molecular shortcuts are available to the phage, then why did we not observe T987C evolve naturally?”* I still do not understand in which experiment or environment it was expected that T987C would evolve. I understand that it is surprising that T987C was evolvable and thermostable but that this came at a cost to growth. The authors could rephrase to make this clearer.

**We have examined this sentence again and understand the confusion, and we have significantly revised this sentence in the manuscript to clarify our intent.**

**In sum, we were attempting to pose the following question: if it is possible to optimize both thermostability and evolvability, as 6-mut T987C seemed to do, why did the ancestral** $\lambda$ **evolve the OmpF innovation via destabilizing mutations in the original experiment, given that instability likely carries a cost? Our finding that 6-mut T987C has a substantially reduced growth rate suggests that there might be a three-way trade-off between thermostability, evolvability, and growth rate. That result could explain why** $\boldsymbol{\lambda}$ **originally evolved the innovation by a more costly route.**

REVIEWER 2

I understand that the authors want to publicize their work by referring to viral emergence and the COVID pandemics. However, this article deals with a very specific phagebacterial system, and extrapolations to the use of receptors in animal viruses are not warranted in this case. Therefore, I suggest refocusing these references (last paragraph, author's abstract). Host shifts are also relevant in the bacterial world, for example for phage therapy. I suggest that the word "emergence" be removed from the title.

Our original response: We have changed the word “emergence” to “host-range” in the title and altered the abstract, author summary, and to more generally discuss virus host-range evolution. However, we would like to push back on the idea that bacteriophages provide no useful insights into animal virus evolution. Leaders in the field of experimental evolution routinely use bacteriophages as model systems to broadly understand the evolution of viruses, and previous papers have discussed how their results provide insights into the evolution of medically relevant viruses (two highly cited examples are listed below). With that in mind, we added a sentence in our final paragraph to make our conclusions more conservative with respect to animal virus applications. “Our results were derived from a study of bacteria-infecting virus rather than an animal virus, and further work is warranted to investigate the role of viral particle instability in animal virus host-range evolvability.” And we also add specific recommendations for how our findings might be applied in this context.

R. C. McBride, C. B. Ogbunugafor, P. E. Turner, Robustness promotes evolvability of thermotolerance in an RNA virus. BMC Evolutionary biology 8: 231 (2008). C. B. Ogbunugafor, R. C. McBride, P. E. Turner. Predicting virus evolution: the relationship between genetic robustness and evolvability of thermotolerance. Cold Spring Harbor Symposia on Quantitative Biology LXXIV, 109-118 (2009).
J. M. Cuevas, A. Moya, R. Sanjuán. A genetic background with low mutational robustness is associated with increased adaptability to a novel host in an RNA virus. J Evol Biol 22, 2041-2048 (2009).

-> OK. Please notice, though, that only the McBride paper was conducted with phages.

**The reviewer is correct- Cuevas et al. was conducted on vesicular stomatitis virus, not bacteriophages. We apologize for the mis-statement. A better example of the prior use of bacteriophages as model systems to study hots-switching and emergence would be:**

**L. Zhao, M. Seth-Pasricha, D. Stemate, A. Crespo-Bellido, J. Gagnon, J. Draghi, S. Duffy. Existing host-range mutations constrain further emergence of RNA viruses. Journal of Virology 93 (2019): e01385-18.**


Finally, the relevance and generality of the conclusions is affected by the fact that, in the model system, the evolutionary trajectory (acquisition of ompF) is rather short (one or few mutations). No other phenotype was examined.

Our first response: We conducted additional experiments in response to this concern. To test the effect of a stabilizing mutation in a background that is further away from the OmpF+ 7-mut, we started with the 6-mut and reverted another mutation, creating a 5-mut. We then edited in the T987A stabilizing mutation, confirmed that it is stabilizing in the 5-mut background, and measured its evolvability relative to 5-mut. We found that, completely in alignment with our model, the stabilizing mutation inhibits evolvability in the 5-mut background. In fact, the difference in evolvability is even greater than that between 6-mut and 6-mut T987A. These results are reported in the last paragraph in the results and Fig 7.

-> This a valuable experiment, but it doesn´t fully address my concern since it focuses on the same trait and evolutionary trajectory. I recommend that the authors add a comment on this to at least acknowledge that the results might potentially be somewhat determined by the specific mechanistic details at play, or may reflect local features of a fitness landscape, rather than reflecting a general process.

**We have carefully gone over the whole document again to identify any places where we have over-generalized our results, and we have edited the author summary and discussion section to de-generalize the statements and add more caveats where appropriate. We would also like to point out multiple places where we had already included caveats to our results and cautioned that our results might not be generalizable:**

**“…our results are based upon a relatively small number of genotypes in a single experimental system, and additional work will be required to assess the generality of the pattern and its use in predicting host-range expansions.”**

**“Our results were derived from a study of bacteria-infecting virus rather than an animal virus, and further work is warranted to investigate the role of viral particle instability in animal virus host-range evolvability.”**

**Unrelated to reviewer comments, we identified and fixed a minor error in Figure 3B. Some of the lines were not scaled correctly. These corrections do not affect interpretation of the figure.**

**Side-by-side comparison of the previous and new version:**
